# National early warning score (NEWS) and the new alternative SpO_2_ scale during rapid response team reviews: a prospective observational study

**DOI:** 10.1186/s13049-019-0691-6

**Published:** 2019-12-16

**Authors:** Joonas Tirkkonen, Sari Karlsson, Markus B. Skrifvars

**Affiliations:** 1 Department of Intensive Care Medicine and Department of Emergency, Anaesthesia and Pain Medicine, Tampere University Hospital, PO Box 2000, FI-33521 Tampere, Finland; 2 Faculty of Medicine, University of Tampere, PO Box 2000, FI-33521 Tampere, Finland; 3 Department of Intensive Care Medicine, Tampere University Hospital, PO Box 2000, FI-33521 Tampere, Finland; 4 Department of Emergency Care and Services, University of Helsinki and Helsinki University Hospital, Helsinki, Finland

**Keywords:** National early warning score, Rapid response team, Respiratory failure

## Abstract

**Background:**

The national early warning score (NEWS) enables early detection of in-hospital patient deterioration and timely activation of hospital’s rapid response team (RRT). NEWS was updated in 2017 to include a separate SpO_2_ scale for those patients with type II respiratory failure (T2RF). In this study we investigated whether NEWS with and without the new SpO_2_ scale for the T2RF patients is associated with immediate and in-hospital patient outcomes among the patients actually attended by the RRT.

**Methods:**

We conducted a two-year prospective observational study including all adult RRT patients without limitations of medical treatment (LOMT) in a large Finnish university associated tertiary level hospital**.** According to the first vital signs measured by the RRT, we calculated NEWSs for the RRT patients and further utilized the new SpO_2_ scale for the patients with confirmed T2RF. We used multivariate logistic regression and area under the receiver operating characteristic analyses to test NEWS’s accuracy to predict two distinct outcomes: RRT patient’s I) immediate need for intensive care and/or new LOMT and 2) in-hospital death or discharge with cerebral performance category >2 and/or LOMT.

**Results:**

The final cohort consisted of 886 RRT patients attended for the first time during their hospitalization. Most common reasons for RRT activation were respiratory (343, 39%) and circulatory (226, 26%) problems. Cohort’s median (Q_1_, Q_3_) NEWS at RRT arrival was 8 (5, 10) and remained unchanged if the new SpO_2_ scale was applied for the 104 patients with confirmed T2RF. Higher NEWS was independently associated with both immediate (OR 1.28; 95% CI 1.22–1.35) and in-hospital (1.15; 1.10–1.21) adverse outcomes. Further, NEWS had fair discrimination for both the immediate (AUROC 0.73; 0.69–0.77) and in-hospital (0.68; 0.64–0.72) outcomes. Utilizing the new SpO_2_ scale for the patients with confirmed T2RF did not improve the discrimination capability (0.73; 0.69–0.76 and 0.68; 0.64–0.71) for these outcomes, respectively.

**Conclusions:**

We found that in patients attended by a RRT, the NEWS predicts patient’s hospital outcome with moderate accuracy. We did not find any improvement using the new SpO2 scale in T2RF patients.

## Background

The national early warning score (NEWS) was first published in 2012 as a simple, feasible tool enabling early detection of in-hospital patient deterioration [1]. Since, it has been widely adopted not just in the United Kingdom, but across European countries, and the usage has spread both to prehospital care and emergency rooms [2–4]. In 2017 the Working Group of the Royal College of Physicians updated the NEWS to include a separate peripheral blood oxygen saturation (SpO_2_) scale for those patients with ‘confirmed hypercapnic respiratory failure (type II respiratory failure, T2RF) on blood gas analysis on either a prior, or their current, hospital admission’ [5]. This updated NEWS, named NEWS2, has only been tested in one retrospective cohort of general ward patients with suspected T2RF, and the results did not support the usage of different SpO_2_ scale with the T2RF patients [6]. However, some debate rose on the retrospective design of the study and the reliability of correct T2RF diagnoses of the patients [7, 8].

Some rapid response team (RRT) patients may be incorrectly triaged to remain on wards; this leads to repeat RRT reviews and possible delays in ICU admittance/ higher morbidity [9, 10]. There are very limited data on the physiological state of the patients that s RRTs actually evaluate on general wards, although vital signs recorded during the reviews could provide important information for system-development, trans-institutional comparisons and data validation. Published data either describe the means or medians of individual vital signs’ measurements or percentages of vital signs with severe deviations from normal range, but these tell very little on the actual status of these patients [11, 12]. However, the NEWS, as a continuous variable constituting of seven different vital signs’ measurements, could provide meaningful description of the status of RRT patients. Further, RRTs encounter frequently patients with respiratory failure [11–13], and a prospective RRT patient cohort could be ideal in studying the NEWS’s new SpO_2_ scale on T2RF patients.

Here we aimed to describe the physiological state of the patients attended by RRT using the first NEWS measured upon RRT arrival. We further investigated NEWSs association with 1) immediate RRT review outcome and 2) patient outcome at hospital discharge. Finally, we studied whether the NEWS2’s new SpO_2_ scale, applied among those RRT patients with confirmed T2RF, increases the accuracy of NEWS to detect patients at risk for adverse outcomes.

## Methods

### Aim and design

To address the above described aims, we designed and conducted a two-year (1.1.2017–31.12.2018) prospective observational single center trial in the Tampere University Hospital (Tays), Finland including all adult RRT reviews. The 2017 updated NEWS2 chart is presented in Additional file 1. Among the normocapnic patients, zero score in the SpO_2_ scale is achieved with percentages ≥96 (with or without supplementary oxygen), whereas among the patients with confirmed hypercapnia the limits for zero score are 88─92% (with or without supplementary oxygen) or ≥ 93% on air.

### Ethics

The Tampere University Hospital’s Ethics Committee approved the study protocol (Approval no: R18203. The Regional Ethics Committee of Pirkanmaa Health District, Tampere University Hospital, PO Box 2000, FI-33521 Tampere, Finland). Patient consent was waived as no interventions were conducted.

### Hospital and RRT

Tays is one of the five university level tertiary referral centres in Finland. With annual 75,000 somatic admissions Tays provides care in all medical specialties for a catchment population of 530,000 people. TAYS has a mixed surgical-medical intensive care unit (ICU) with 20 beds and approximately 2000 admissions per year, and a separate ICU with six beds for cardiothoracic surgery patients.

Hospital’s RRT operates 24/7 from the ICU, and includes an ICU physician and two RRT-trained ICU nurses. Our RRT was implemented in 2009 with dichotomized RRT activation criteria (heart rate < 40/min or > 140/min, systolic blood pressure < 90 mmHg, SpO_2_ < 90%, respiratory rate < 5/min or > 24/min, decrease in state of consciousness and the ‘worried’ criterion) which are still in use, although the general ward staff have been encouraged to utilize the NEWS since our program to implement this early warning score begun in 2017. The RRT responds to all medical emergencies, including in-hospital cardiac arrest calls.

### Data collection and definitions

We collected prospectively data on all RRT activations between 1.1.2017–31.12.2018 using specialized RRT review templates that included all the components suggested by the 2007 Ustein Statement [14]. During the RRT reviews, the ICU nurses measured all vital signs as usual, but also calculated the NEWS from the first measured vital signs at RRT arrival. Data on the RRT reviews were inputted to a Microsoft® Excel Worksheet, and in this phase the NEWSs were recalculated with an electronic application by the primary investigator to ensure that scores were correctly recorded. Additional data regarding patient- and hospital admission characteristics were collected from the electronic patient records. We used the Charlson comorbidity index to describe the burden of comorbidities before the current admission, and the cerebral performance category (CPC, based on hospital discharge day’s physician-, physiotherapist- and nursing notes) to describe patients’ neurological status at hospital discharge [15, 16]. For those patients that had confirmed T2RF according to criteria declared in the NEWS update 2017 in their patient notes (confirmed hypercapnic respiratory failure on arterial blood gas analysis (PaCO_2_ > 6 kPa) on either a prior, or their current, hospital admission [5]), we re-calculated additional NEWS2 utilizing the new SpO_2_ scale.

### Outcomes

We aimed to investigate NEWS’s association (with and without the NEWS2’s new SpO_2_ scale for those patients with T2RF) with two distinct outcomes. The immediate RRT review outcome was a combined outcome of whether a patient was immediately transferred to ICU and/or new limitation of medical treatment (LOMT) was issued as an RRT review result for a previously LOMT-free patient. We hypothesized that this outcome would describe accurately the need for intensive care (or withdrawal from intensive care procedures due to futility), as at least in our hospital the RRT usually implements new LOMT only when the team would otherwise consider admitting the patient to the ICU. The in-hospital outcome was a combined outcome of whether a patient either died in hospital or was discharged from the hospital with new LOMT and/ or with poor neurology suggested by a CPC of 3 or higher. We hypothesized that this combined outcome would describe the unfavorable patient outcome more accurately than just the raw hospital mortality rate.

### Exclusion criteria

Only the first RRT activations were included from those patients that required multiple RRT attendances during their hospitalization. RRT activations for paediatric patients (< 18 years) were excluded, and we also excluded all RRT activations concerning patients already in the ICU. RRT activations due to or resulting in cardiac arrests were excluded. Finally, patients with preceding LOMT were excluded from the analyses.

### Statistical analyses

Data are presented as numbers (percentages) unless otherwise indicated. We used multivariate logistic regression analysis with the ‘enter’ method to investigate those factors independently associated with the previously described immediate and in-hospital outcomes. Hosmer-Lemeshow tests were conducted to present the goodness-of-fit data for the multivariate models. The area under the receiver operating characteristic (AUROC) analysis was used to test the discriminative performance of the NEWS on the two outcomes. We considered *p* <  0.05 significant and 95% confidence intervals were reported where appropriate. We used SPSS version 20 for Windows (SPSS Inc., Chicago, IL, USA) for the statistical analyses.

## Results

### Study cohort

**Fig. 1:**
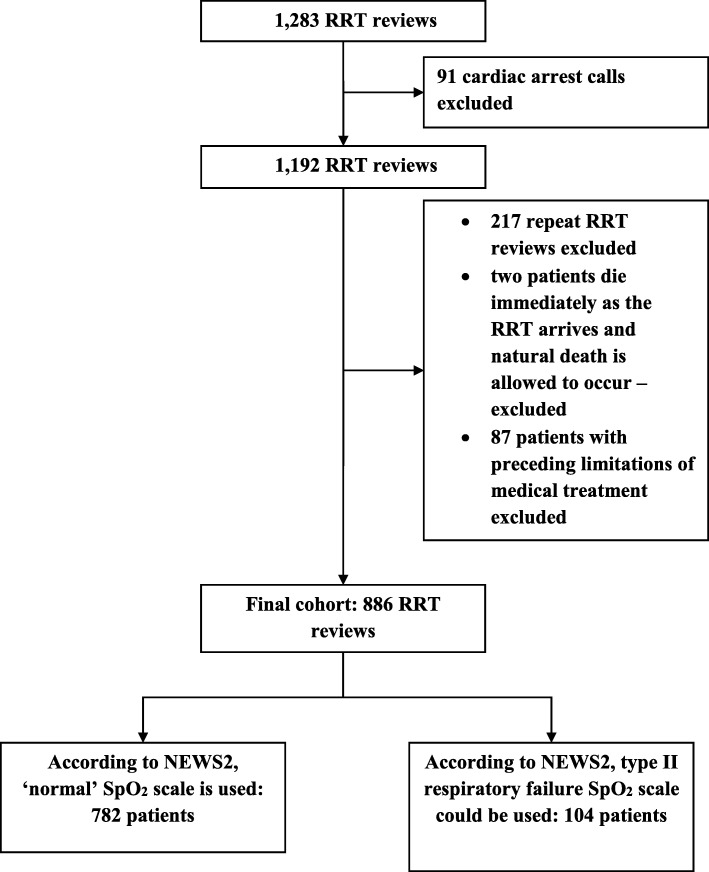
Rapid response team activations during the two-year study period and the final cohort. RRT, rapid response team; NEWS2, the updated national early warning score; SpO_2_, peripheral blood oxygen saturation

There were 1283 RRT reviews during the two-year trial period. Figure 1 presents in detail the excluded cases and the final cohort of 886 RRT patients. Table 1 presents the patient characteristics, admission and RRT review characteristics, and the outcomes among the study cohort. There were 104 patients that fulfilled the T2RF definition according to the patient notes and arterial blood gas results.

**Table 1:** Study cohort characteristics

Patient demographics	
Age (years), median (Q_1_,Q_3_)	70 (60, 78)
Gender (male)	492 (56)
CPC 1–2 before the current admission	877 (99)
Performance in daily living; lives home independently	710 (80)
Charlson comorbidity index, median (Q_1_,Q_3_)	2.0 (0, 3)
COPD	73 (8.2)
Diabetes	244 (28)
Malignancy	237 (27)
Dementia	44 (5.0)
PAD	74 (8.4)
Coronary artery disease	132 (15)
Hospital admission characteristics	
Elective hospital admission	258 (29)
Days in hospital before the RRT activation median (Q_1_, Q_3_)	2 (1, 5)
Surgical diagnosis for admission	519 (59)
Preceding ICU admission during hospitalization	91 (10)
Surgery conducted 0─24 h before	93 (11)
RRT review characteristics	
On-call time RRT activation	233 (26)
Reason for RRT activation	
Respiratory	343 (39)
Circulatory	226 (26)
Decreased mental status	225 (25)
Nurse worried	39 (4.4)
Other	53 (6.0)
RRT interventions	
Supplementary oxygen started or flow changed	89 (10)
HFNOT/CPAP/BiPAP started	103 (12)
Endotracheal intubation	17 (1.9)
Intravenous fluids	191 (22)
Blood products	56 (6.3)
Medications	278 (31)
Continuous vital signs’ monitoring started on ward	110 (12)
Transfer to ICU	171 (19)
ICU mortality (% of the transferred patients)	17 (10)
Transfer to PACU/OR	46 (5.2)
Transfer to emergency department	53 (6.0)
New limitations of medical treatment issued	66 (7.4)
Hospital outcome	
Died	134 (15)
Discharged alive	752 (85)
CPC at discharge 1–2	697 (79)
CPC at discharge 3–4	55 (6.2)
Limitations of medical treatment at discharge	111 (13)
Discharged directly to home	252 (28)

Data are presented as numbers (percentages) if not otherwise indicated. *CPC* Cerebral performance category, *COPD* Chronic obstructive pulmonary disease; malignancy, malignant solid tumor, lymphoma or leukemia according to the ICD-10, International Statistical Classification of Diseases and Related Health Problems 10th Revision (C00-C97, ICD-10), *PAD* Peripheral arterial disease, *RRT* Rapid response team, *ICU* Intensive care unit, *HFNOT* High-flow nasal oxygen therapy, *CPAP* Continuous positive airway pressure, *BiPAP* Bilevel Positive Airway Pressure, *PACU* Post anaesthetic care unit, *OR* Operating room

The median (Q1, Q3) NEWS during the RRT reviews was 8 (5, 10) among the whole cohort, and this result did not change if the analysis was conducted applying the NEWS2’s new SpO_2_ scale for the 104 patients with T2RF background. NEWSs’ range among the cohort was 0–20. Four fifths (711, 80%) of the cohort had NEWS >4 and 535 patients (60%) scored >6 (median and high scores according to the NEWS). If just the 104 patients with T2RF were assessed separately their median NEWS was 9 (7.25, 12) and 9 (6, 11) if the NEWS2’s new SpO_2_ scale was applied.

### NEWS in the multivariate regression model

**Fig. 2:**
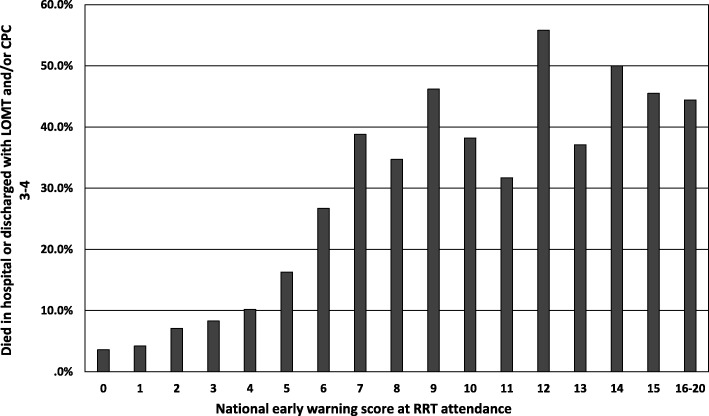
Scores 16–20 are presented in one bar as only five patients had NEWS >16. LOMT, limitation of medical treatment; CPC, cerebral performance category; RRT, rapid response team

Figure 2 shows how the incidence of adverse outcomes at hospital discharge increased as higher NEWSs were observed. After adjusted for eight covariates potentially affecting patient outcome during hospitalization, higher NEWS was independently associated with both immediate (OR 1.28; 95% CI 1.22–1.35) and in-hospital (1.15; 1.10–1.21) adverse outcomes (Table 2).

**Table 2:** Multivariate logistic regression analysis of factors independently associated with immediate and in-hospital outcomes of RRT patients

	Multivariate analysis		
Transfer to ICU or new LOMT	Odds ratio	95% CI	*p*-value
Age	1.00	0.99–1.01	0.92
Non-elective hospital admission	0.92	0.62–1.37	0.69
CCI	1.03	0.94–1.12	0.58
Sex (female)	0.77	0.60–1.07	0.13
Medical patient	0.95	0.67–1.35	0.77
Surgery 0–24 h before the review	0.70	0.39–1.27	0.24
Preceding ICU admission	1.11	0.65–1.88	0.70
National early warning score	1.28	1.22–1.35	<0.01
Review during on-call time^a^	1.03	0.70–1.50	0.90
Died in hospital or discharged with LOMT and/or CPC 3–4			
Age	1.04	1.03–1.015	< 0.01
Non-elective hospital admission	2.93	1.92–4.47	< 0.01
CCI	1.11	1.02–1.21	0.02
Sex (female)	1.08	0.78–1.48	0.65
Medical patient	1.14	0.82–1.59	0.45
Surgery 0–24 h before the review	0.99	0.56–1.75	0.98
Preceding ICU admission	1.30	0.76–2.22	0.34
National early warning score	1.15	1.10–1.21	< 0.01
Review during on-call time^a^	1.02	0.71–1.47	0.93

The Hosmer-Lemeshow goodness-of-fit Chi-squares (7.84, *p* = 0.45) and (13.2, *P* = 0.10) indicated a good fit for both the models. *RRT* Rapid response team, *ICU* Intensive care unit, *LOMT* Limitations of medical treatment, *CI* Confidence interval, *CCI* Charlson comorbidity index, *CPC* Cerebral performance category. ^a^On-call time: Other than Monday − Friday 7.30 a.m. to 3.00 p.m.

### Performance of NEWS and the new SpO_2_ scale to predict immediate and in-hospital adverse outcomes

**Fig. 3:**
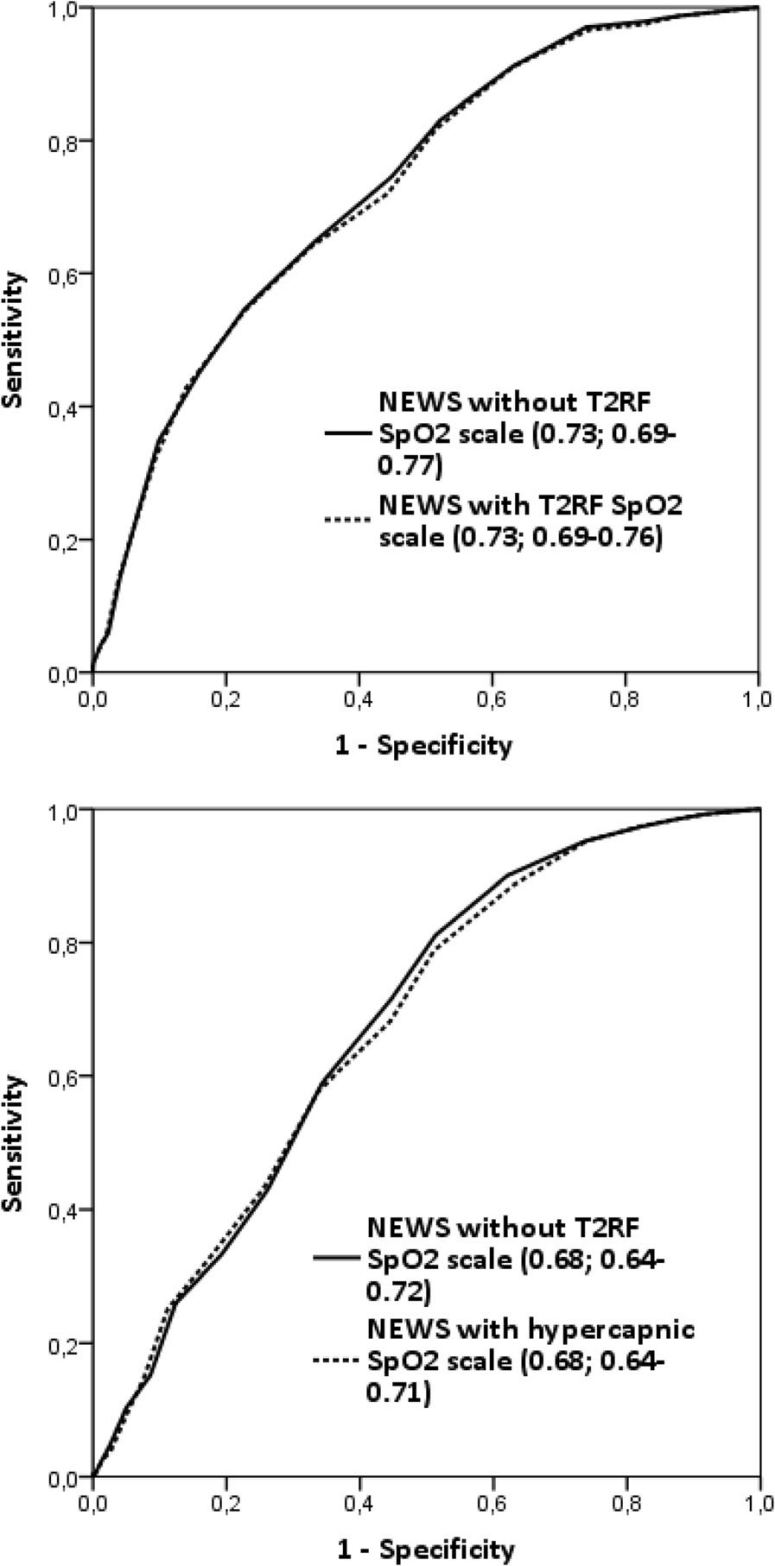
NEWS’s AUROC analyses for immediate (upper) and in-hospital (lower) adverse outcomes - the whole cohort. NEWS, national early warning score; AUROC, area under the receiver operating characteristic; T2RF, type 2 respiratory failure; SpO_2_, peripheral blood oxygen saturation

**Fig. 4:**
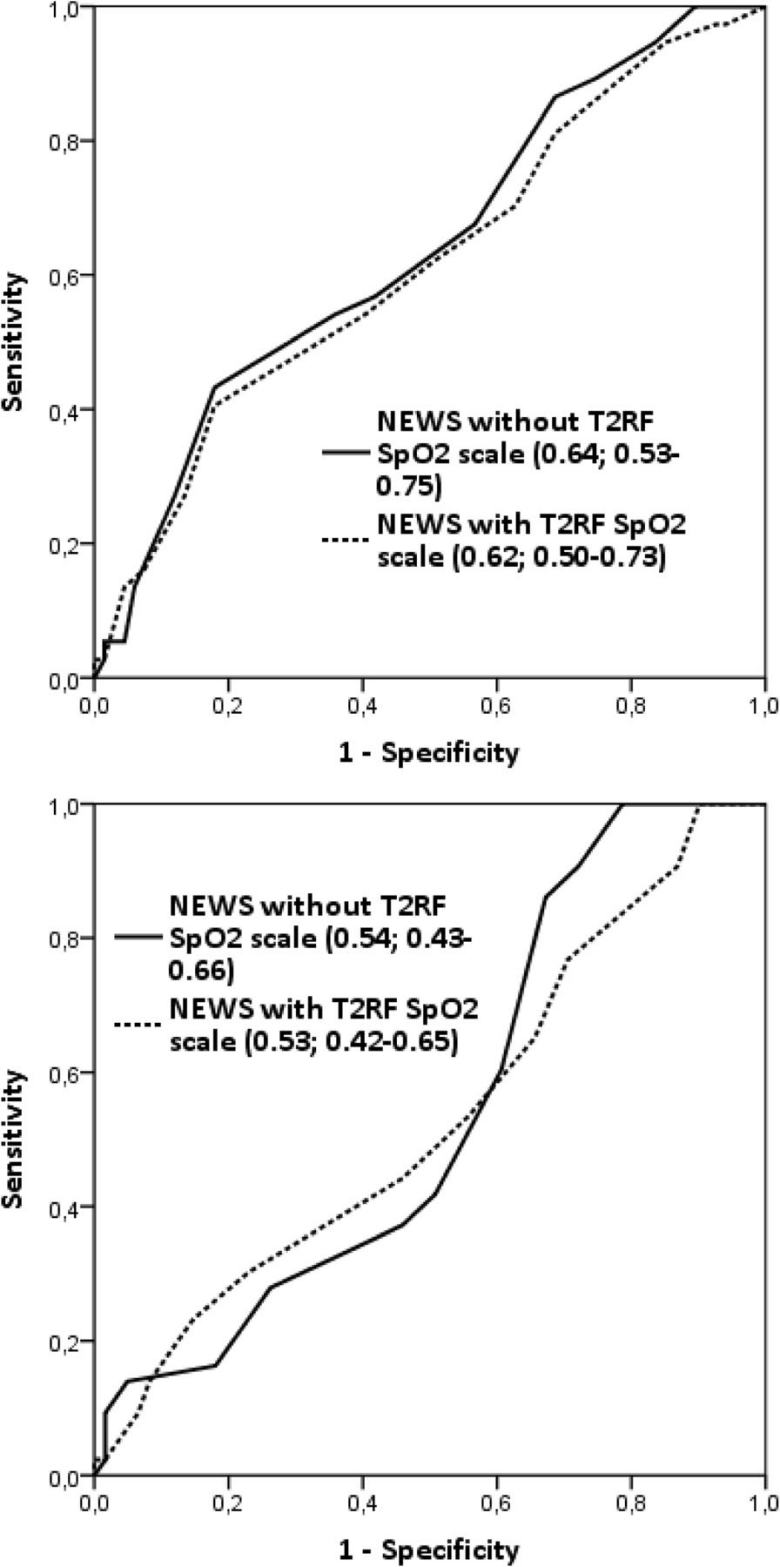
NEWS’s AUROC analyses for immediate (upper) and in-hospital (lower) adverse outcomes - the T2RF sub-cohort. NEWS, national early warning score; AUROC, area under the receiver operating characteristic; T2RF, type 2 respiratory failure; SpO_2_, peripheral blood oxygen saturation

NEWS had a fair ability to predict both immediate (AUROC 0.73; 0.69–0.77) and in-hospital (0.68; 0.64–0.72) adverse outcomes **(**Fig. 3). The performance did not change if the new SpO_2_ scale for the T2RF patients was used (Fig. 3). If the NEWS was tested among just the 104 T2RF patients (Fig. 4), the AUROC indicated poor discrimination for immediate adverse outcome and no discrimination for in-hospital adverse outcome; utilizing the new SpO_2_ scale did not improve these results.

## Discussion

### Key findings

To our knowledge, this is the first large prospective trial that 1) describes the overall physiological state of RRT patients at the time of RRT attendance with an early warning score and 2) compares NEWS’s predictive performance with NEWS’s updated SpO_2_ scale for those patients with actual type 2 respiratory failure. We found that 80% of the RRT patients scored NEWS seven or more, which is the key trigger threshold for emergency assessment and underlines the need for higher level of care. Higher RRT NEWS was independently associated with immediate- and in-hospital adverse outcomes with acceptable statistical discriminative performance. These results suggest the applicability of NEWS not only for initiation of a RRT but also for identification of the patient at risk at time of the call. We did not, however, find any benefit from applying the new SpO_2_ scale in the type 2 respiratory failure patients regarding the identification of the patient at risk.

### Strengths and limitations of the study

This study was conducted in a large university-level tertiary center with mature rapid response system and was of prospective design. Further, the NEWS values were carefully double-checked during the process with a computer software to guarantee that the scores were correctly recorded (studies have repeatedly shown how early warning scores are miscalculated by the nursing staff) [17, 18]. We excluded patients who already had LOMT as critical care interventions were ruled out and in many cases severely deviating vital signs are just preceding death that is allowed to occur among these patients. Most importantly, in our study we meticulously reviewed the patient records and laboratory result-sheets in order to correctly identify patients with type 2 respiratory failure according to the NEWS development group (patients with confirmed hypercapnic respiratory failure on arterial blood gas analysis (PaCO_2_ > 6 kPa) on either a prior, or their current, hospital admission) [5]. Finally, we used patient centered outcomes rather than just classifying ‘being alive’ (survival) as a good outcome.

This study was conducted among patients already under RRT review and indeed this is a limitation as NEWS was originally developed to enable early detection of patient deterioration on general wards. On the other hand, most in-hospital studies on NEWS have been conducted among emergency department cohorts and actual general ward cohorts are rare. Nevertheless, as both the incidence of adverse events and the prevalence of confirmed T2RF are substantially higher as compared with general ward cohorts, our results must be interpreted with these facts in mind. Further, more clear limitations of internal validity include the issues that 1) some wards used both NEWS criteria and the old dichotomized criteria to activate the RRT and 2) the AUROC analysis including just the 104 T2RF patients must be reviewed with extreme caution due to small number of cases. External validity of our results is limited to larger hospitals with similar organizational structure and ability to treat the most complex patient groups.

### Condition of the patients attended by the RRT

Whilst several studies have documented the vital signs (and abnormalities in vital signs) among general ward patients, there is actually very little data on the results of the vital signs’ measurements when RRTs review patients whose condition is considered to be deteriorating according to the ward staff [17, 19, 20]. Coventry et al. reported that no-LOMT RRT patients took median 20 breaths/min and had median 120mmhg systolic blood pressure at RRT arrival, and we have previously reported that 38% of RRT patients had respiratory rate < 5 or > 24 /min and 14% had systolic blood pressure < 90mmhg at RRT arrival [11, 12]. These few examples, however, demonstrate how difficult it is to deduce the overall state of homeostasis of these RRT patients from average statistical values of individual vital signs. The NEWS provides a comprehensive picture on the actual status of a patient, as all vital signs are included in a single, continuous variable [5]. Several studies have reported NEWS values among out-of-hospital, emergency room and general ward patient cohorts. Hoikka et al. reported a median NEWS of 2 (range 0–18) among 12,000 prehospital patients, while Alam et al. reported a median NEWS of 2 (range 0–11) among emergency room patients which was indeed comparable to that of our previously documented emergency room cohort (median 2, range 0–15) [3, 4, 21].

On the other hand, sub cohort studies have reported much higher average NEWSs; De Groot et al. documented a median NEWS of 6 (interquartile range 4–9) among geriatric emergency room patients with suspected infection, while Sbiti-Rohr et al. found that 37% of the emergency room patients with community acquired pneumonia had NEWS of 7 or more [22, 23]. In our hospital, median NEWS among unselected general ward patients was just 1 (0, 3) in 2010 [24]. Comparing our current results to the existing literature, it is clear that the RRT evaluates patients far more unstable as compared even with the more comorbid and severely ill patients in emergency rooms. Indeed, our RRT patients seem to have higher median NEWSs as compared with those patients who are transferred directly to ICU from our emergency room with median NEWS of 7 (3, 9) [21]. This is the first study to record the NEWSs among RRT patients, but we suggest that in future research the NEWS could provide vital information on how rapid response systems function in different institutions. Today, the efficiency of rapid response systems still remain somewhat controversial because so many unknown confounding factors exist between different RRT studies [25].

### NEWS predictive performance among the RRT patients

NEWS had a fair discriminate performance for adverse outcomes among the RRT patients. In different cohorts NEWSs discriminative performance has varied from AUROCs of 0.78–0.88 to 0.65–0.67 depending on the study settings [5, 21–23]. Perhaps the key question is, what do we want to predict? In studies, early warning scores are without exception tested against different adverse outcomes (24 h mortality, in-hospital mortality, 30-day mortality) and there has been justified criticism against these kind of study settings, as with NEWS we simply aim to identify in time those patients that are at risk of dying but salvageable if we act on time [7, 8]. However, it is practically impossible to determine correctly those patients that did not die because of an intervention triggered by an early warning score and use it as an outcome without an obvious risk for the Hawthorne effect [26]. Keeping these facts in mind, we focused on patient centered outcomes. Whilst it is true that being discharged with new limitations of medical treatment is not an adverse outcome for all patients, it describes how the hospital admission ultimately ended with withdrawal from invasive interventions. Currently there are no validated triage tools for the RRTs to be utilized during a patient review, although the study group by Cardona-Morrell et al. have introduced the CriSTAL tool to be used among elderly gerastenic RRT patients [27]. With our results we suggest that since NEWS is statistically valid among the RRT patients, RRTs could also themselves use the NEWS during the reviews. Indeed, multiple NEWS measurements during the reviews would provide simple but critical information on whether the conducted interventions are improving patient’s condition. Again, further studies are warranted on this topic since no preceding data exists.

### NEWS and the new SpO_2_ scale for the patients with documented T2RF

In early 2019 the study group behind the development and validation of the original NEWS published a retrospective validation study of the new Spo2 scale for the T2RF patients. Unfortunately, identifying the patients fulfilling the T2RF criteria was based on assumptions drawn from the International Statistical Classification of Diseases and Related Health Problems-10 coding [6]. The results did not support the usage of a different Spo2 scale depending on the patients’ respiratory background. This result is to be taken seriously, as every additional item in any scoring system increases the risk for miscalculations and misinterpretations. Interestingly, however, Echevarria et al. found in their study of 2600 emergency room patients with spirometry-proven chronic obstructive pulmonary disease (COPD) (not hypercarbic proven as required in the updated NEWS) that best statistical performance was achieved if the new SpO_2_ was applied for all COPD patients, not just those with the T2RF [28]. The discriminative performance was poorest, when just the ‘normal’ SpO_2_ scale was applied for all patients in this COPD cohort [28]. On the other hand, Hodgson et al. studied quite a similar cohort of 942 emergency room patients with acute exacerbation of COPD and found the original NEWS to have fair (and substantially better as compared with the study by Echevarria et al.) discriminative performance for in-hospital mortality [28, 29]. The problem is, that these studies consisted of sub cohorts (COPD patients), whereas the premise of the NEWS is to provide nursing staff with a simple scoring system that is feasible for all adult patients [1]. Our results suggest that applying the new SpO_2_ scale strictly for those patients with the NEWS development group definition for T2RF, no beneficial effects are seen in statistical discriminative performance. Granted, our cohorts consist of the most severely ill ward patients, but on the other hand our cohort includes general ward patients from all medical specialties, some post-operative, some post-ICU patients and with different reasons for possible T2RF. In light of our current results, using the new SpO_2_ scale strictly for those patients with ‘confirmed hypercapnic respiratory failure on blood gas analysis on either a prior, or their current, hospital admission’ is not well-grounded, and indeed the obvious limitations related to the requirement of arterial blood gas analysis before even considering the usage of the new SpO_2_ scale seriously questions the feasibility of the system.

## Conclusions

RRT patients have higher NEWSs as compared with the trigger thresholds meant to prompt RRT reviews and in general most RRT patients have severely disturbed overall physiology according to NEWS. NEWS has fair discriminative capability for adverse outcomes among RRT patients and thus could be used by the RRT members as a triage tool to identify the patients at highest risk for morbidity/mortality benefiting from immediate ICU admission. Our results do not support any benefit from the new SpO_2_ scale of NEWS in type 2 respiratory failure patients.

## Supplementary information

**Additional file 1.** The updated National early warning score (NEWS2) according to Royal college of Physicians. SpO2 Scale 2 is intended to be used among patients with confirmed type II respiratory failure. SpO2, peripheral blood oxygen saturation; Alert (A), (C) New Confusion, Voice (V), Pain (P), Unresponsive (U).

## Data Availability

The data of the current study are not publicly available as the Ethics Committee’s approval restricts redistribution of any data. However, upon a well-justified reason a permission to review pseudonymized data could be applied from the Tays Ethics Committee.

## Abbreviations

AUROC: Area under the receiver operating characteristic
CI: Confidence interval
COPD: Chronic obstructive pulmonary disease
ICU: Intensive care unit
LOMT: Limitation of medical treatment
NEWS: National early warning score
OR: Odds ratio
RRT: Rapid response team
SpO_2_: peripheral blood oxygen saturation
T2RF: Type 2 respiratory failure
TAYS: Tampere University Hospital
