# Supplementary material for: National early warning score (NEWS) and the new alternative SpO2 scale during rapid response team reviews: a prospective observational study
Source: Scand J Trauma Resusc Emerg Med. 2019 Dec 16;27:111. doi: 10.1186/s13049-019-0691-6 (PMC6915867; doi:10.1186/s13049-019-0691-6)
Supplement: Supplementary file 1 — Additional file 1. The updated National early warning score (NEWS2) according to Royal college of Physicians. SpO2 Scale 2 is intended to be used among patients with confirmed type II respiratory failure. SpO2, peripheral blood oxygen saturation; Alert (A), (C) New Confusion, Voice (V), Pain (P), Unresponsive (U). [file 13049_2019_691_MOESM1_ESM.docx]

Additional file 1.

| Score | 3 | 2 | 1 | 0 | 1 | 2 | 3 |
| --- | --- | --- | --- | --- | --- | --- | --- |
| Respiratory rate (breaths/min) | ≤8 |  | 9─11 | 12─20 |  | 21─24 | ≥25 |
| SpO2 Scale 1 | ≤91 | 92─93 | 94─95 | ≥96 |  |  |  |
| SpO2 Scale 2 | ≤83 | 84─85 | 86─87 | 88─92  ≥93 on air | 93─94 | 95─96 | ≥97 on oxygen |
| Any supplementary oxygen |  | Yes |  | No |  |  |  |
| Temperature (*C) | <35.0 |  | 35.1─36.0 | 36.1─38.0 | 38.1─39.0 | ≥39.1 |  |
| Systolic blood pressure (mmHg) | ≤90 | 91─100 | 101─110 | 111─219 |  |  | ≥220 |
| Heart rate (beats/min) | ≤40 |  | 41─50 | 51─90 | 91─110 | 111─130 | ≥131 |
| A, C, V, P, U |  |  |  | A |  |  | C,V, P, U |

SpO_2_ Scale 2 is intended to be used among patients with confirmed type II respiratory failure. SpO_2_, peripheral blood oxygen saturation; Alert (A), (C) New Confusion, Voice (V), Pain (P), Unresponsive (U).
